# 1H NMR Based Targeted Metabolite Profiling for Understanding the Complex Relationship Connecting Oxidative Stress with Endometriosis

**DOI:** 10.1155/2013/329058

**Published:** 2013-08-05

**Authors:** Saikat K. Jana, Mainak Dutta, Mamata Joshi, Sudha Srivastava, Baidyanath Chakravarty, Koel Chaudhury

**Affiliations:** ^1^School of Medical Science and Technology, Indian Institute of Technology, Kharagpur 721302, India; ^2^National Facility for High-Field NMR, Tata Institute of Fundamental Research, Mumbai 400005, India; ^3^Institute of Reproductive Medicine, Kolkata 700106, India

## Abstract

Accumulating evidence indicates the active role of oxidative stress in the development of endometriosis; however, the mechanism of reactive oxygen species generation is poorly understood. Metabonomics/metabolomics is a scientific discipline that can be used to study changes in metabolite ensembles associated with disease pathophysiology. The present study focuses on the use of proton nuclear magnetic resonance spectroscopy based targeted metabolite profiling approach to explore dysregulation in metabolites expression in women with endometriosis. Further, association of oxidative stress with the metabolite ensembles, if any, is investigated. Using multivariate statistics, partial least square discriminant analysis model was generated which could classify endometriosis patients with sensitivity and specificity of 92.83% and 100%, respectively, and with a classification rate of 96.4%. In conjunction with increased glucose metabolism, citrate and succinate were found to be elevated in endometriosis patients. Higher levels of reactive oxygen species, lipid peroxidation, and advanced oxidation protein products and lower levels of total antioxidant capacity, superoxide dismutase, catalase, and glutathione were also observed. Increased glucose metabolism and defects in the mitochondrial respiratory system are suggested to be the possible sources of excessive reactive oxygen species generation in endometriosis.

## 1. Introduction

Endometriosis is classically defined as the growth of endometrial glands and stroma at extrauterine sites, most commonly implanted over visceral and peritoneal surfaces within the female pelvis. In India, approximately 1% women undergoing major gynecological surgery, 6%–43% undergoing sterilization, 12%–32% undergoing laparoscopy for pelvic pain, of 21%–48% undergoing laparoscopy for infertility are diagnosed with endometriosis [1]. From a global perspective, it is a fairly common gynecological disorder affecting almost 10% of women of reproductive age [2]. The correct approach for endometriosis management is still unclear. The risks and the diagnostic limitations of laparoscopy and the inaccuracy of clinical examination justify the considerable efforts made to improve the diagnosis with noninvasive techniques. In our previously published paper, we have reported a metabolic fingerprint which may be used for non-invasive diagnosis of the disease [3]. Also, the therapeutic approach still focuses on management of clinical symptoms of the disease rather than on the disease itself. A thorough understanding of the pathophysiology of endometriosis is therefore essential to the development of novel diagnostic and treatment approaches for this debilitating condition.

Several early studies in 1990s showed no significant correlation between oxidative stress (OS) and endometriosis [4–6]. Murphy and coworkers, way back in late 1990s, were one of the first groups to emphasize on the active role of OS in the pathogenesis and development of endometriosis [7]. Since then there has been increasing evidence suggesting that OS plays a key role in the pathogenesis and progression of endometriosis [8, 9]. Recently, a systematic review of 19 original articles from 1990 to 2011 on OS biomarkers in patients with endometriosis has been reported [10]. Despite such long-term research activity in this field, it is still not very clear as to when and why OS may occur and its relation to endometriosis, possibly because of limited data and complex environment of the peritoneal cavity. 

During OS, in addition to insufficient antioxidants, oxygen radicals are also formed at a rate greater than the rate of consumption. Changes in the composition of metabolite ensembles are responsible for physiological changes associated with OS. Thus, it is reasonable to conjecture that targeted metabolite profiling will provide information on quantitative changes in metabolites of interest based on prior knowledge of the biological function or metabolic pathway. 

Metabolomic profiling has emerged as a powerful and reliable tool for the identification of total metabolites present in the biological system under a given physiological condition. Metabolites represent the final products of cells' regulatory processes and act as communicators between information-rich genome and the functional phenotype of the cell. Nuclear magnetic resonance (NMR) spectroscopy is the only technique which can identify and quantify complex mixtures of metabolites with little or no sample preparation [11]. Furthermore, only small sample volumes are required, and the analysis is nondestructive [12]. An added advantage of this technique is that the metabolite profile of a biological sample can be acquired rapidly (1–15 min) with sufficient sensitivity to differentiate even subtle biological differences. Chemometrics and targeted profiling are the two distinct approaches that have been established for processing NMR spectra. Chemometrics involves separation of NMR spectra into different groups with no assumptions about the identity and quantity of metabolites in the spectra while targeted profiling involves the identification and quantification of each metabolite in every NMR spectrum, so that metabolite concentrations are the variables [13]. In both the approaches, various multivariate statistical methods such as principal components analysis (PCA) partial least squares discriminant analysis (PLS-DA) are used to search for meaningful differences among the spectra.

Herein, we have used 1H NMR based targeted metabolite profiling followed by multivariate statistical analysis to explore metabolite imbalances, which in turn, would provide a better understanding of oxidative protection, injury, and recovery in endometriosis. Further, association of OS with the metabolite ensembles, if any, is investigated, and a probable mechanism of ROS generation in endometriosis is hypothesized.

## 2. Material and Methods

### 2.1. Subject Selection and Sample Collection

One hundred and thirty five women (24–40 years, BMI < 25) reporting at the Institute of Reproductive Medicine, Salt Lake, Kolkata, India, for infertility treatment volunteered to participate in this study. The study group consisted of 75 women with endometriosis confirmed by diagnostic laparoscopy, and 60 women with tubal factor infertility were considered as controls. Tubal factor infertility refers to women who had salpingectomy for ectopic pregnancy and proximal tubal obstruction because of low-grade infection or fimbrial occlusion with or without mild peritubal adhesions. Tubal infertility associated with gross hydrosalpingeal changes, dense pelvic adhesions because of endometriosis or pelvic inflammatory diseases were excluded. As such, in this study, tubal factor infertility refers to women who had fallopian tube(s) removed for tubal pregnancy. Women included in the study did not receive any kind of medical or hormonal treatment during the last three months. Women with history of removal of chocolate cysts, previous history of any kind of gynecological surgery including lower pelvic and abdominal surgery, with other possible causes of pain or pelvic pathology including pelvic tuberculosis were excluded. The study was approved by the Institutional ethics committee, and written informed consent was taken from all women enrolled in the study.

Venous blood was drawn in sterile containers from all women during the day time in fasting state and in their early follicular phase. Based on the effects of estrogen, it was hypothesized that the magnitude of the responses would be greater when measured during the early follicular phase compared to the rest of the menstrual cycle. The blood were then allowed to clot and serum separated by centrifugation at 3,000 rpm for 5 min at 4°C. ROS was measured immediately in the serum of all subjects. Remaining serum samples were stored at −20°C until further analysis for total antioxidant capacity (TAC), lipid peroxidation (LPO), superoxide dismutase (SOD), glutathione (GSH), and catalase levels. Further, 26 samples from endometriosis cases and 24 from controls were randomly selected from these stored serum samples and used for metabolic profiling using NMR. 

### 2.2. Measurement of Reactive Oxygen Species

Free radicals generation was determined in serum samples from endometriosis and controls by monitoring luminol (5-amino-2,3-dihydro-1,4-phthalazinedione)-mediated chemiluminescence (CL) [14]. Luminol reacts with reactive oxygen species (ROS) present in serum resulting in a luminophore that has an emission peak at ~425 nm. The intensity of the CL is proportional to the amount of ROS in the serum. The reaction mixture contained 25 *μ*L of serum and 0.1 mM luminol in 10 mM sodium phosphate buffer (pH 7.4). The reaction was initiated by the addition of H_2_O_2_ at a final concentration of 1 mM. CL was measured for 10 min using a luminometer (Berthold, Sirius Single tube Luminometer, Model no. 0727). H_2_O_2_ was added as an activator of luminol. Background (blank) was determined in each experiment utilizing H_2_O_2_, luminol, and sodium phosphate buffer without samples. The reaction was performed at 37°C and expressed in relative light units (cps).

### 2.3. Measurement of Lipid Peroxidation

The slightly modified thiobarbituric acid (TBA) method [15] was used to measure malonaldehyde (MDA) content in the serum sample. Fifty microliter aliquot of frozen serum was thawed and immediately used for lipid peroxidation (LPO) estimation. Hundred microliter of stock reagent (12%, w/v trichloroacetic acid, 0.375%, w/v TBA and 0.25 mol/L HCl warmed to dissolve the TBA) was mixed thoroughly with 50 *μ*L of the sample and heated for 15 min in a boiling water bath. After cooling, the flocculent precipitate was removed by centrifugation at 1000 ×g for 10 min and the OD of the supernatant determined at 535 nm in multiplate reader (Victor X3, Perkin Elmer, USA) against a blank containing all the reagents. LPO values were expressed as *μ*M MDA.

### 2.4. Measurement of Total Antioxidant Capacity

 TAC was measured in serum using a modified enhanced CL assay [16]. Serum was thawed at room temperature and diluted 1 : 10 with deionized water. Signal reagent was prepared using a CL kit (GE Healthcare, UK). A constant peak of ROS was produced using HRP conjugated IgG (Santa Cruz Biotechnology, Santa Cruz, CA). HRP was diluted with deionized water to give a desired CL output. Luminometer was set in the kinetic mode, and 900 *μ*L of reaction mixture (100 *μ*L signal reagent + 700 *μ*L deionized water + 100 *μ*L 1 : 350 diluted HRP) was added to the cuvette. After 100 seconds of reaction mixture addition, 50 *μ*L of the diluted serum sample was added to the mixture, and 10% recovery of CL was recorded. Trolox, a water soluble tocopherol analogue, was used as a standard. The antioxidant capacity of serum samples was expressed in *μ*M Trolox equivalents.

### 2.5. Determination of the Superoxide Dismutase Enzymatic Activity

Serum superoxide dismutase (SOD) activity was measured utilizing the inhibition of auto-oxidation of pyrogallol by SOD [17]. Tris buffer (containing 50 mM of Tris buffer and 1 mM of ethylene diamine tetraacetic acid, EDTA) was prepared, and pH was adjusted to 8.5 using hydrochloric acid. One hundred microliter of 20 mM pyrogallol solution was added to 2.9 mL of Tris buffer and mixed for controls measurement. Reading was taken at 420 nm after 1 min 30 s and 3 min 30 s using a UV-visible spectrophotometer (Perkin Elmer, USA). Serum SOD activity was measured by adding 0.1 mL of diluted serum sample to 2.8 mL of Tris buffer. The reaction was started by adding 0.1 mL of 20 mM pyrogallol solution. Reading was taken at 420 nm exactly after 1 min 30 s and 3 min 30 s and the absorbance recorded per 2 min. The percentage of inhibition of pyrogallol autoxidation was calculated, and 1 unit of enzymatic activity was defined as the quantity of enzyme necessary to achieve a 50% inhibition of autoxidation at 25°C.

### 2.6. Determination of the Catalase Enzymatic Activity

The activity of catalase enzyme was determined according to Aebi [18]. The capacity of catalase to transform H_2_O_2_ was measured by monitoring the decomposition of H_2_O_2_ at 240 nm. The final reaction volume of 3 mL contained 0.05 M Tris-buffer, 5 mM EDTA (pH 7.0), and 10 mM H_2_O_2_ (in 0.1 M potassium phosphate buffer, pH 7.0). Fifty microliter of serum sample was added to the above mixture. One unit of enzymatic activity was considered as the quantity of enzyme necessary to transform 1 *μ*mol of H_2_O_2_ in 1 min at 37°C. Catalase activity was calculated using the molar extinction coefficient of 0.0436 (mmol L^−1^)^−1^ cm^−1^ and expressed in terms of mM H_2_O_2_ consumed/min/mg protein used.

### 2.7. Determination of the Glutathione

Glutathione (GSH) in the serum samples was estimated by the method of Moron et al. [19]. The required amount of serum was mixed with 25% of trichloroacetic acid (TCA) and centrifuged at 2000 ×g for 15 min to settle the precipitated proteins. The supernatant was aspirated and diluted to 1 mL with 0.2 M sodium phosphate buffer (pH 8.0). Later, 2 mL of 0.6 mM 5,5′-dithiobis-2-nitrobenzoic acid (DTNB) was added. After 10 min, the OD of the yellow-colored complex formed by the reaction of GSH and DTNB was measured at 405 nm. A standard curve was obtained with standard GSH. The levels of GSH were expressed as *μ*mol/mL.

### 2.8. Determination of Advanced Oxidation Protein Products

Determination of advanced oxidation protein products (AOPP) was based on spectrophotometric detection according to Witko-Sarsat et al. [20]. Two hundred microliter of serum diluted 1 : 5 in PBS, 200 *μ*L of chloramine-T standard solution (0 to 100 *μ*mol/liter) for calibration, and 200 *μ*L of PBS as blank were placed in each well of a 96-well microliter plate (PerkinElmer, USA). Ten microliter of 1.16 M potassium iodide (KI, Sigma) was then added, followed by 20 *μ*L of acetic acid. The absorbance of the reaction mixture was immediately read at 340 nm in a microplate reader against a blank. The chloramine-T absorbance at 340 nm was linear within the range of 0 to 100 *μ*mol/liter. AOPP concentrations were expressed in *μ*mol/liter of chloramine-T equivalents.

### 2.9. Nuclear Magnetic Resonance Analysis

Prior to nuclear magnetic resonance (NMR) analysis, serum samples were thawed and homogenized using a vortex mixer. Two hundred microliter of serum was mixed with 400 *μ*L deuterium oxide (D_2_O) containing 1 mM sodium salt of 3-(trimethylsilyl)propionic-2,2′,3,3′,d4 acid (TSP). After centrifugation (8000 rpm, 5 min), 600 *μ*L of each sample was transferred to 5 mm NMR tubes. Proton NMR spectra were recorded at 298 K using a 700 MHz Bruker Avance AV III spectrometer. The resulting spectra were phased and baseline corrected by Bruker TOPSPIN 2.1 software. Individual metabolites were identified from various sources, including earlier published articles and literature, and cross checked from the Human Metabolome Database (HMDB).

### 2.10. Statistical Analysis

Two types of statistical analysis were performed for analysis of the entire data presented in this paper.

#### 2.10.1. Student *t*-Test

Data were analyzed using analysis of *t*-test, as appropriate. Data analyses were performed with (GraphPad QuickCalcs, 2002–2005, GraphPad Software, Inc., USA). Statistical significance was defined as *P* ≤ 0.05.

#### 2.10.2. Multivariate Statistical Analysis

Expression of different metabolites in endometriosis compared to controls was analyzed using multivariate statistical analysis. The analysis was applied to a total of 50 spectra from 26 endometriosis and 24 control serum samples. After acquisition, data matrix of peak integral values corresponding to identified compounds was built. Data were preprocessed using normalization and scaling to remove possible bias arising due to sample handling and sample variability. Normalization (by sum) was performed in order to minimize possible differences in concentration between samples. Following normalization, scaling (mean-centering and division by the square root of standard deviation of each variable) was done to give all variables equal weight regardless of their absolute value. After data preprocessing, PCA and PLS-DA were performed using web-based metabolomic data processing tool MetaboAnalyst 2.0 (Canada) (accessible at http://www.metaboanalyst.ca/) [21]. In MetaboAnalyst, statistical computing and visualization operations are performed using functions from the R and Bioconductor packages [22, 23]. PCA is used to detect intrinsic clusters and outliers within the data set, while PLS-DA maximizes class discrimination. Model robustness was assessed using another web-based ROC curve analysis tool, ROCCET (accessible at http://www.roccet.ca/). ROC curves in ROCCET are generated by Monte Carlo Cross Validation (MCCV) [24] using balanced subsampling. In each MCCV, two-third (2/3) of the samples were used to evaluate the feature importance. The top 100 important features were then used to build classification models which were validated on one-third of the samples that were left out. The procedures were repeated multiple times to calculate the performance of the PLS-DA model. Sensitivity and specificity of the model were calculated from the confusion matrix generated from the multiple MCCV iterations. Further validation was performed with MetaboAnalyst 2.0, using permutation tests consisting of 1000 permutations.

#### 2.10.3. Metabolic Pathway Analysis

Detailed analysis of the most relevant metabolic pathways and networks in women with endometriosis was performed by MetaboAnalyst that combines results from powerful pathway enrichment analysis involved in the conditions under study. MetaboAnalyst uses high-quality KEGG metabolic pathways as the back end knowledge base. It integrates many well-established methods (i.e., univariate analysis, over-representation analysis), and novel algorithms and concepts (i.e., Global Test, Global-Ancova, network topology analysis) with pathway analysis [25].

## 3. Results

**Figure 1:**
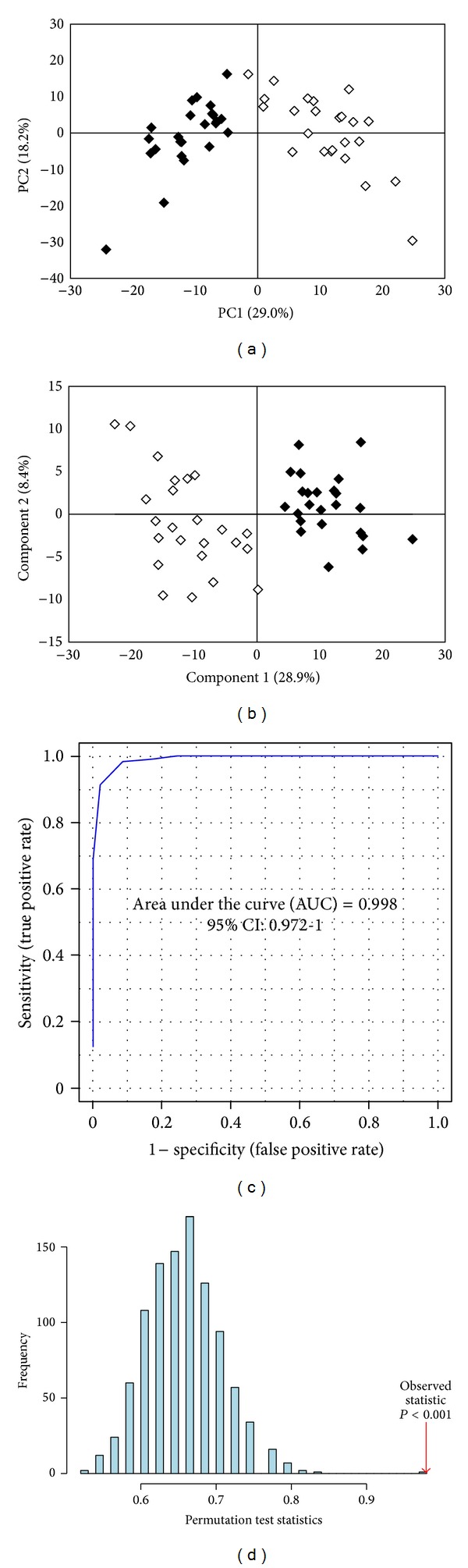
Scores scatter plot of (a) PC1 versus PC2 and (b) component 1 versus component 2 resulting from PCA and PLS-DA, respectively, applied to 1H NMR spectra of serum of endometriosis patients (black diamond) and controls (white diamond). Validation of the PLS-DA model was performed by (c) receiver operating characteristic (ROC) analysis where area under the curve (AUC) was found to be 0.99 (d) Permutation test statistics at 1000 permutations with observed statistic at *P* < 0.001.

OS markers in serum of women with endometriosis and controls are summarized in Table 1. ROS, LPO, and AOPP were observed to be increased significantly whereas TAC, SOD, catalase, and GSH levels were significantly less in endometriosis women as compared to controls. Several amino acids, organic acids, and other molecules were identified in serum using 1H NMR metabolic profiling. A representative metabolic fingerprint of serum from women with endometriosis is shown in Supplementary Figure 1 in Supplementary Materials available online at http://dx.doi.org/10.1155/2013/329058. Exploratory PCA of identified metabolites was employed to detect intrinsic clustering and possible outliers.  Figure 1(a) depicting PC1 versus PC2 scores scatter plot shows a trend for unsupervised separation between endometriosis and controls. PLS-DA further maximized the group separation (Figure 1(b)). Leave one out cross-validation (LOOCV) was employed from which *Q* ^2^ and *R* ^2^ values (representing the predictive capability and the explained variance, resp.) were extracted. The model with *R* ^2^ close to 0.83 and *Q* ^2^ well above 0.82 showed a very good predictive ability. To better assess the predictive ability of the PLS-DA classification model, MCCV was applied as discussed in Section 2. Sensitivity (percentage of endometriosis samples correctly classified as true positives) and specificity (percentage of control samples correctly classified as true negatives) of the PLS-DA model were found to be 100% and 91.67%, respectively, with a classification rate (total number of samples correctly classified) of 95.83%. Area under the ROC curve (Figure 1(c)) was found to be 0.99, denoting high predictive accuracy of the model. Further, model validation relied on permutation analysis. The analysis was performed only for the best model. If the performance score of the original data lies outside the distribution, then the result is significant. Using 1000 permutation tests, the *P* value is reported as *P* ≤ 0.001, denoting that none of the results are better than the original one (Figure 1(d)). The further away from the plot origin a variable lies, the stronger impact the variable has on the model. From PLS-DA loading plot, metabolites with higher loadings were identified (Supplementary Figure  2). On comparing the loading and scores plot, it becomes evident that lactate, 2-hydroxybutyrate, succinate, lysine, glycerophosphocholine, citric acid, pyruvate, adipic acid and lipids are overexpressed in endometriosis whereas isoleucine, arginine, asparagine, glucose, creatine, alanine, leucine and fatty acid have higher expression in controls. Signals with high VIP values (Supplementary Figure 3) are considered to be significant and therefore validated using *t*-test (Table 2). With pattern recognition analysis of profiles of metabolites, a clear separation between endometriosis and controls was achieved. As a consequence of dysregulation of specific metabolites, metabolic pathway analysis was performed using MetaboAnalyst. The affected pathways (Impact-value ≥ 0.10) in women with endometriosis are represented in Supplementary Figure 4.

## 4. Discussion

The present study is an attempt to measure levels of various OS parameters in serum of endometriosis patients and identify differently expressed metabolites using 1H-NMR based targeted profiling to have an improved understanding of the disease pathophysiology.

Several studies have reported ROS concentration to be higher in women with endometriosis [6, 26] which is in agreement with our findings. Endometriosis has also been associated with significantly higher levels of lipid peroxide-modified rabbit serum albumin, malondialdehyde-modified low-density lipoprotein, and oxidized low-density lipoprotein as measured in serum and compared to tubal ligation cases [27]. Furthermore, lipid peroxide concentrations have been reported to be highest in women with endometriosis indicating the involvement of ROS in the development of the disease [9]. These observations support the results of the present study where significant increase in LPO levels in endometriosis women is observed. 

Catalase, SOD and GSH are utilized to keep ROS in check. It is thus imperative that lower levels of these enzymes would indicate increased ROS production which is evidenced by the findings of the present study (Table 1). A significant decrease in TAC level in endometriosis women was also observed in the present study (Table 1). This is in accordance with the findings of Szczepańska and coworkers who have documented that mean activity of SOD and total antioxidant status are lowest among infertile patients with endometriosis [9]. Ota et al. reported a higher expression of SOD in endometrial tissues of endometriotic women as compared to controls by immunohistochemistry, which is a semiquantitative method [28]. On the contrary, Szczepańska et al. observed reduced SOD level in peritoneal fluid in endometriosis as compared to controls using quantitative spectrophotometric assay [9]. This is in accordance with our present study, where reduced SOD level were observed in serum of endometriotic women. From the present study, OS condition is indicated in endometriosis, as evidenced by lower expression of TAC, SOD, GSH, catalase, and higher ROS generation.

 Protein cross-linking products formed due to oxidation of amino acids by ROS in the plasma are designated as AOPP [29]. AOPP are formed during OS by the action of chlorinated oxidants, mainly hypochlorous acid and chloramines [30]. AOPP are predominantly aggregates of albumin damaged by OS [29]. Increased levels of AOPP in endometriosis observed in the present study are in agreement with increased OS reported in these women.

Multivariate analysis of the identified NMR spectra clearly distinguishes endometriosis cases from the controls. On plotting PC1 (29.0%) × PC2 (18.2%), the plots for endometriosis are observed to lie in a principal component plane which is significantly different from the plane where plots for controls are clustered (Figure 1(a)). As evidenced in Figure 1(b), PLS-DA shows statistically significant separation between endometriosis and control cases. Our values corresponding to *R* ^2^ and *Q* ^2^ close to 0.8 indicate that the model is valid and can predict better than chance. Sensitivity, specificity and classification rates of 100%, 91.67%, and 95.83%, respectively, further validate the model robustness. Also, permutation test statistics result (*P* < 0.001) validates the predictive ability of the real model. Metabolites with high VIP scores including lactate, L-alanine, glycerophospholcholine, glucose, L-leucine, L-lysine, creatine, L-arginine, succinic acid, adipic acid, lipid, pyruvate, 2-hydroxybutyrate, L-isoleucine, L-asparagine, and citric acid were considered as significant.

**Figure 2:**
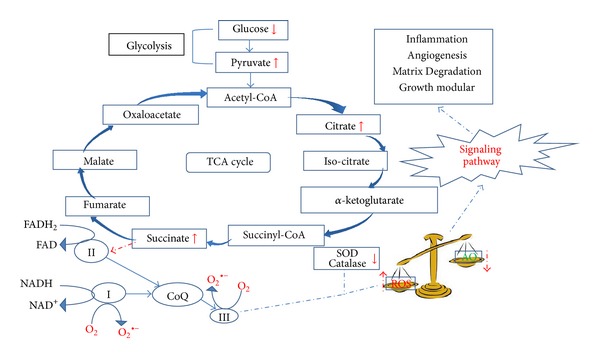
Figure depicting the role of metabolites in generation of free radicals. Lower levels of glucose along with elevated levels of pyruvate, citrate, and succinate indicate enhanced glucose metabolism along with impaired mitochondrial respiration. I, II, and III represent complex I, II, and III, respectively, of electron transport chain. Complex I and III are the primary sites of free radical generation. Elevated levels of succinate indicate complex III to be the most probable site for ROS generation in endometriosis. These free radicals are further removed from the system by various ROS scavengers such as SOD and catalase. An imbalance in the rate of formation and removal of free radicals results in oxidative stress. In endometriosis, lower levels of these enzymes lead to continued prevalence of oxidative stress.

The tripeptide glutathione is reported to be critical in the detoxification of ROS [31]. Glutathione is considered as one of the most abundant antioxidants present in cells [32, 33]. Reduced glutathione donates electron to ROS and gets oxidized, thus helping in scavenging unstable ROS. Increased GSSG/GSH ratio is indicative of OS [34]. This is in agreement with our results which indicate a significant decrease in GSH. Ophthalmate has been indicated as a biomarker of OS as insufficient levels of GSH results in ophthalmate synthesis [35]. 2-hydroxybutyrate is released as a by-product during ophthalmate synthesis. Metabolite profiling of serum revealed increased levels of 2-hydroxybutyrate which further support the prevalence of OS in endometriosis. Unlike ROS detoxifying agents like SOD and catalase that only decompose ROS without having an impact on their production, the TCA cycle can regulate both, their formation and decomposition [35]. Glucose levels were found to be significantly less in endometriosis as compared to controls. Increased metabolism of glucose in conjunction with up-regulation of pyruvate suggests enhanced glycolysis in these women. It is well known that all aerobic organisms rely predominantly on the TCA cycle to generate NADH and FADH2 from acetyl CoA for the synthesis of ATP during oxidative phosphorylation. Other than the production of ATP, mitochondria are also involved in the cell's response to OS. Several steps in the path of oxygen reduction in mitochondria have the potential to produce highly reactive free radicals that can damage cells. 0.1%–4% of the O_2_ used by actively respiring mitochondria forms ^●^O_2_^−^ which can have lethal effects on a cell unless the free radical is quickly disposed of. In the present study, in conjunction with increased glucose metabolism, citrate and succinate, two TCA cycle intermediate products, were also found to be elevated in endometriosis patients. These data support the notion that a possible defect is associated with the mitochondrial respiratory system with an increased probability of ROS generation from the ETC. A probable hypothesis summarizing the possible route of free radical generation in endometriosis patients is depicted in Figure 2. While in normal cells various antioxidants dispose of ROS from the system, insufficient levels of SOD and catalase in endometriosis are indicative of inefficient scavenging effect leading to a continued prevalence of ROS in the system.

Leucine and isoleucine are essential amino acids. Leucine catabolism terminates at acetyl CoA whereas isoleucine gives rise to acetyl CoA and propionyl CoA. Moreover, acetyl CoA provides the carbon atoms of its acetyl group to the TCA cycle for energy production. Decreased serum levels of both leucine and isoleucine indicate enhanced catabolism of these amino acids leading to elevated TCA cycle intermediates. 

Adipic acid is a byproduct of LPO which is nonenzymatically mediated by ROS. NMR metabonomic data indicating a significant decrease in lipid and increase in adipic acid levels supported by the higher levels of LPO estimated biochemically reconfirms the involvement of OS. L-lysine is an essential amino acid which is known to reduce OS [35]. Since lysine uses the same intracellular transporter system as arginine, hence lysine competes with arginine for its transport within the cells. Elevated lysine and lower arginine levels in serum of women in endometriosis may be attributed to increased uptake of arginine by the cells. 

Glycerophosphocholine (GPC) plays a pivotal role as an osmotic pressure regulator and metabolic antitoxin. It is a major reservoir for cell membrane omega-3 phospholipids, the major building blocks for cell membranes. It helps in supporting membrane fluidity, enabling membrane proteins to perform with better efficiency. Under OS condition, GPC is reported to get oxidized [36] and reduced GPC levels in serum may lead to reduced cell membrane fluidity and efficiency.

Asparaginase is an enzyme which converts asparagine into ammonia and aspartate. Aspartate transaminates to oxaloacetate, which follows the gluconeogenic pathway to form glucose. Lower serum levels of gluconeogenic amino acids including asparagine and alanine in endometriosis may be due to its increased utilization as a major gluconeogenic precursor, to meet the high glucose uptake and demand by fast growing endometrial cells outside the uterus. 

## 5. Conclusion

This is the first attempt to identify the potential causes of ROS generation in women with endometriosis. NMR based targeted metabolic profiling method was used for this purpose. The preliminary findings suggest increased glucose metabolism and defects in the mitochondrial respiratory system to be possible sources of excessive ROS generation in these women. It may be argued that these preliminary results are not reliable enough to functionally associate OS with metabolite pathways. Follow-up laboratory efforts leading to experimental validation targeted specifically to the predicted pathway are, therefore, necessary.

## 6. Limitation

Multivariate statistical analysis is useful in providing association between multiple risk factors and disease condition. It was, therefore, used to identify the potential causes of ROS generation in endometriosis cases. However, these preliminary results require further validation to functionally associate OS with the proposed metabolite pathway.

## Supplementary Material

A representative 1H NMR spectrum of serum obtained from a woman with endometriosis, PLS-DA loading and VIP plot, and the pathway analysis of the metabolites identified are provided.

## Figures and Tables

**Table 1:** Levels of oxidative stress parameters in endometriosis and controls.

Parameters	Endometriosis (*n* = 60)	Controls (*n* = 60)	*P* value
ROS (cps)	129.5 ± 1.9	73.84 ± 1.7	*P* ≤ 0.001
LPO (*μ*M MDA)	1.366 ± 0.04372	1.014 ± 0.03146	*P* ≤ 0.001
TAC (*μ*M Trolox equivalent)	733.6 ± 4.989	936.3 ± 4.141	*P* ≤ 0.001
SOD (units/mL)	23.19 ± 0.43	39.23 ± 0.48	*P* ≤ 0.001
Catalase (nmol/min/mg)	30.63 ± 0.7389	49.09 ± 1.148	*P* ≤ 0.001
GSH (*μ*mol/mL)	3.06 ± 0.06898	7.01 ± 0.08175	*P* ≤ 0.001
AOPP (*μ*mol/L)	155.1 ± 4.250	90.60 ± 3.445	*P* ≤ 0.001

Mean ± SEM.

**Table 2:** Main serum metabolites contributing towards discrimination between endometriosis and controls.

Metabolites	*δ* 1H (ppm)	Fold changes (relative to controls)
Glucose	3.26	0.26
Alanine	1.5	0.32
Creatine	3.06	0.13
2-hydroxybutyrate	3.96	1.55
Lactate	4.11	1.92
L-lysine	3.04	1.72
Pyruvate	2.47	1.58
Succinic acid	2.39	1.48
Adipic acid	1.54	1.61
L-leucine	0.99	0.51
Citric acid	2.54	1.67
L-iso leucine	1.03	0.82
Glycerophosphocholine	3.66	1.85
L-asparagine	2.84	0.41
L-arginine	1.64	0.94

## Abbreviations

OS: Oxidative stress
ROS: Reactive oxygen species
LPO: Lipid peroxidation
OD: Optical density
TBA: 3-(methyl thio)butyric acid
MDA: Malondialdehyde
TAC: Total antioxidant capacity
HRP: Horseradish peroxidase
SOD: Superoxide dismutase
GPC: Glycerophosphocholine
GSH: Glutathione
AOPP: Advanced oxidation protein products
NMR: Nuclear magnetic resonance
D_2_O: Heavy water.
