# Supplementary material for: 1H NMR Based Targeted Metabolite Profiling for Understanding the Complex Relationship Connecting Oxidative Stress with Endometriosis
Source: Biomed Res Int. 2013 Aug 5;2013:329058. doi: 10.1155/2013/329058 (PMC3747613; doi:10.1155/2013/329058)
Supplement: Supplementary file 1 — A representative 1H NMR spectrum of serum obtained from a woman with endometriosis, PLS-DA loading and VIP plot, and the pathway analysis of the metabolites identified are provided. [file 329058.f1.doc]

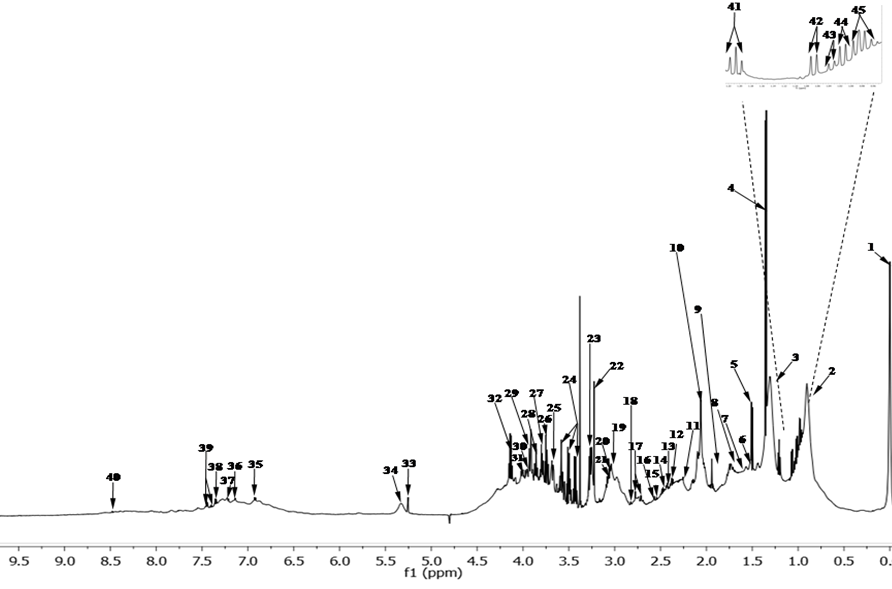


Supplementary Figure 1: A typical 700 MHz 1H-NMR spectrum of serum from an endometriosis patient. Numbers indicate the following metabolites: 1,TSP ; 2, lipid; 3, fatty acid; 4, lactate ; 5, alanine; 6, adipic acid; 7, L-arginine; 8 & 45, L- leucine; 9, acetate; 10 & 43, L-isoleucine; 11, acetone; 12, 26, 42 & 44, valine; 13, succinic acid 14, L-glutamine; 15, pyruvate; 16, citric acid; 17, L- aspartate;18, L-asparagine; 19, L-lysine; 20 & 31, creatine; 21, ornithine; 22, choline; 23, 24, 28 & 33, D- glucose; 25, threonine; 27, glycerophosphatidylcholine; 29, 2-hydroxybutyrate; 30 & 36, L-histidine; 32, lactate; 34, urea; 35 & 37, tyrosine; 38, imidazole; 39, L-phenylalanine; 40, formate; 41, 3-hydroxybutyric acid.


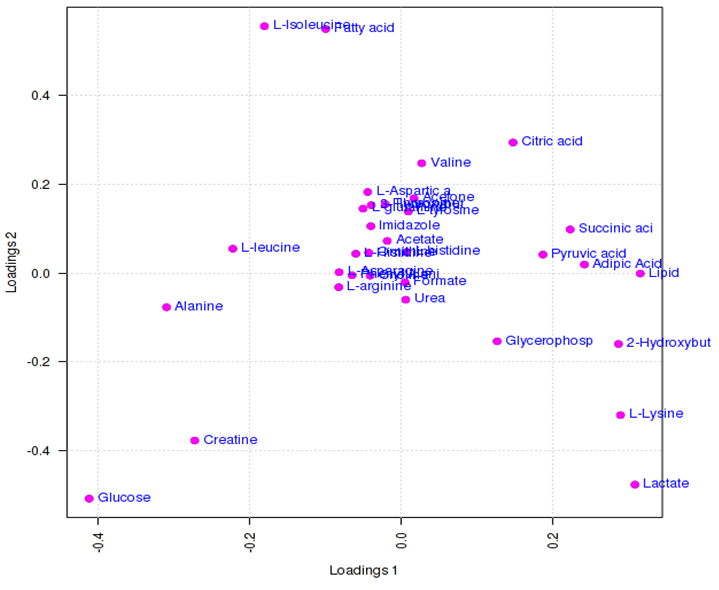


Supplementary figure 2: PLSDA loading plot for component 1 and 2 metabolites


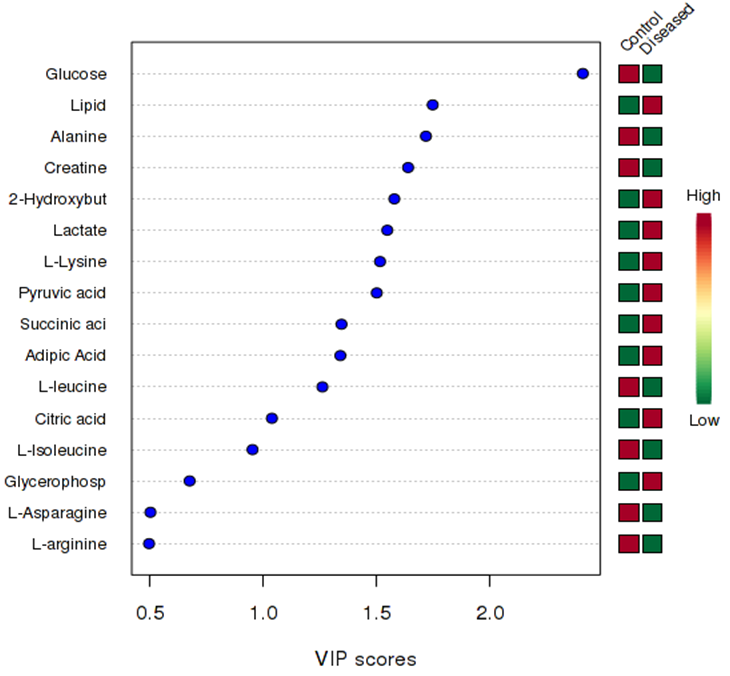


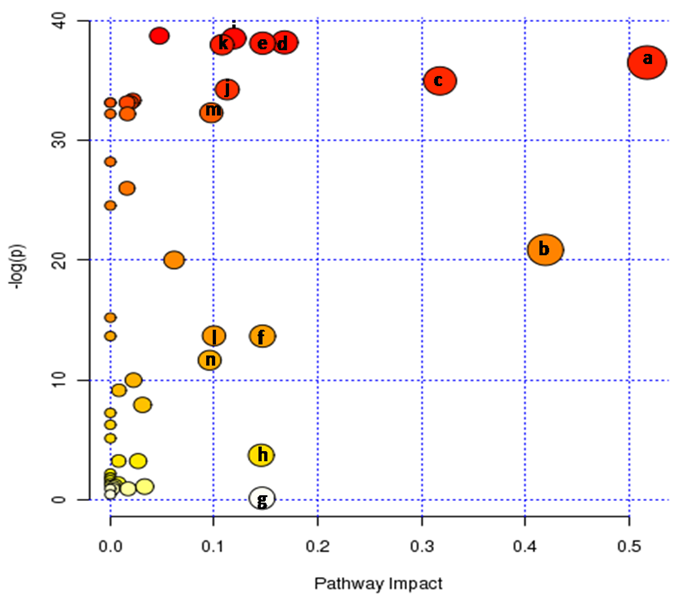
Supplementary figure 3: Important features identified by PLS-DA and VIP scores. The colored boxes on the right indicate the relative integral of the corresponding spectra in each group under study

**Supplementary figure 4:** Summary of pathway analysis with Metaboanalyst. (a) Alanine, aspartate and glutamate metabolism (b) Pyruvate metabolism (c) Arginine and proline metabolism (d) Citrate cycle (TCA cycle) (e) Glyoxylate and dicarboxylate metabolism (f) Lysine degradation (g) Methane metabolism (h) Histidine metabolism (i) Phenylalanine metabolism (J) Aminoacyl-tRNA biosynthesis(k) Butanoate metabolism (l) Lysine biosynthesis (m) Glycine, serine and threonine metabolism (n) Glycolysis or Gluconeogenesis
